# Experimental and Theoretical Insights on Methylene Blue Removal from Wastewater Using an Adsorbent Obtained from the Residues of the Orange Industry

**DOI:** 10.3390/molecules26154555

**Published:** 2021-07-28

**Authors:** Stephanie Giraldo, Irma Robles, Luis A. Godínez, Nancy Acelas, Elizabeth Flórez

**Affiliations:** 1 Grupo de Investigación Materiales con Impacto (Mat&mpac), Facultad de Ciencias Básicas, Universidad de Medellín, Carrera 87 No. 30-65, Medellín 050026, Colombia; stepha930925@hotmail.com; 2 Centro de Investigación y Desarrollo Tecnológico en Electroquímica S. C., Parque Tecnológico Querétaro, Sanfandila, Pedro Escobedo 76703, Querétaro, Mexico; irobles@cideteq.mx (I.R.); lgodinez@cideteq.mx (L.A.G.)

**Keywords:** adsorption, dyes, orange peel, adsorption mechanism, DFT

## Abstract

Chemical and thermochemical transformations were performed on orange peel to obtain materials that were characterized and further tested to explore their potential as adsorbents for the removal of methylene blue (MB) from aqueous solutions. The results show the high potential of some of these materials for MB adsorption not only due to the surface area of the resulting substrate but also to the chemistry of the corresponding surface functional groups. Fitting of the kinetic as well as the equilibrium experimental data to different models suggests that a variety of interactions are involved in MB adsorption. The overall capacities for these substrates (larger than 192.31 mg g^−1^) were found to compare well with those reported for activated carbon and other adsorbents of agro-industrial origin. According to these results and complementary with theoretical study using Density Functional Theory (DFT) approximations, it was found that the most important adsorption mechanisms of MB correspond to: (i) electrostatic interactions, (ii) H-bonding, and (iii) π (MB)–π (biochar) interactions. In view of these findings, it can be concluded that adsorbent materials obtained from orange peel, constitute a good alternative for the removal of MB dye from aqueous solutions.

## 1. Introduction

Contamination of water bodies by the continuous discharge of wastewater containing dyes from textile, paper, cosmetic and, pharmaceutical industries [1,2], have caused a deterioration of aquatic ecosystems and, methylene blue (MB) is one of the most harmful basic/cationic dyes, used for dyeing products such as wood, silk, and cotton [3,4,5]. 

Reducing the organic dye contaminations is of great importance due to the inherent toxicity of the dye molecules and because these compounds decrease light penetration within water, thus all of the photosynthetic processes are seriously affected. Therefore, several organic dye reduction techniques to remove the dye component from the wastewater have been developed. Adsorption compared to other treatment techniques, such as, chemical deposition, reverse osmosis, coagulation, and flocculation [6,7] has been identified as a superior technology due to its low cost, high efficiency, ease of operation, wide variety of available adsorbents which can be used repeatedly for many cycles and proves to be very cost-effective [8]. 

Adsorbents produced from agro-industrial wastes are considered as an interesting alternative due to their high accessibility, low production cost, high porosity, variety of surface functional groups, and high efficiency for dye removal [9,10,11]. In fact, it will help to solve the problem of agro-industrial waste disposal through the development of a circular economy with the efficient valorization of these wastes.

In Colombia, citrus juice companies discard between 15 and 25 tons of orange peel (OP) where only a fraction is employed to extract pectin, limonene [12] and, biopolymers used in the food and pharmaceutical industry [13]. The remaining residue is frequently disposed without any further use. Chemical and physical modifications of OP allow one to design adsorbent materials for the efficient removal of organic contaminants, such as MB. For example, heat treatments at low temperatures allow one to obtain adsorbent materials rich in oxygenated functional groups [14] and low surface areas [15]; while the use of chemical activating agents allows for modification of both the surface area and the quantity and variety of surface functional groups. For example, ZnCl_2_ allows for generation of adsorbent materials with high surface area due to dehydration and elimination of the most volatile biomass components [16] while H_3_PO_4_ allows one to generate a surface rich in oxygenated functional groups [17], such as carboxylic, lactonic, and phenolic groups, which have been reported as active in the MB adsorption process. The combination of different biomass modification processes allows obtaining materials with varied physicochemical properties to be applied in the elimination of pollutants. Several authors have reported good adsorption properties for the material adsorbent produced from OP to remove MB from water with adsorption capacities ranging from 7.57 to 218 mg g^−1^ or thermally transformed materials and for chemically transformed materials [18,19,20,21]. These properties are comparable with the ones reported for commercial activated carbons, where the maximum adsorption capacities are from 45 to 195 mg g^−1^ [13,19,21,22,23,24]. 

The main drawback is that most of the studies are focused on determining adsorption capacities [25] and characterizing the process through the application of kinetic and isotherm models [26,27], and very few studies [28,29] have been reported where computational chemistry tools are also combined to understand the adsorption mechanism and the role of functional groups present in the materials used. For instance, in their study, Sellaoui et al. [29] found that the hydrogen and oxygen functionalities of biomass surface were the major in charge functional groups for dye adsorption. Density functional theory (DFT) has become a powerful and informative tool to: (i) assess the atomic understanding of the adsorption mechanism; (ii) achieve the design of a “functionalized” material to remove cationic dyes; and (iii) predict the feasibility of adsorption of a specific adsorbent targeting a particular adsorbate. The molecular understanding of these interactions and, the influence of pH on the MB removal process, are important parameters because it will allow the understanding of the adsorption mechanism and the optimization of the process conditions not only for dye removal, but for the subsequent regeneration of the adsorbent and eventually, for the design of other novel engineered adsorbent surfaces.

Thus, the novelty of the present work was the systematic transformation of biomass to produce adsorbent materials of different nature and, the characterization of the interactions between MB and the adsorbents by using the experimental data related with density functional theory (DFT) calculations. 

## 2. Methodology

### 2.1. Materials and Transformation Methods

**Figure 1:**
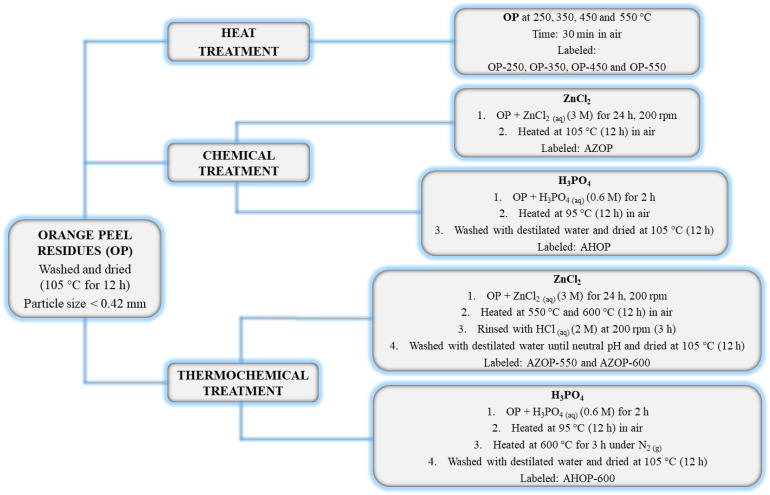
Orange peel transformation methods to obtain adsorbent materials.

Figure 1 presents a summary of the different methods of orange peel transformations.

Orange peel (OP) for this study was collected after consuming orange juice. The residue was washed several times with distilled water and dried in an oven at 105 °C for 12 h. The material was then cut into small pieces, grounded (particle size < 0.420 mm) and stored. OP biomass was modified by thermal, chemical, and thermochemical transformations. Initially, the OP biomass was transformed by heat treatment at different temperatures using an oven set at temperatures of 250, 350, 450, and 550 °C for 30 min. The resulting materials were labeled OP-250, OP-350, OP-450, and OP-550, respectively. Chemical transformation was performed using ZnCl_2_ and H_3_PO_4_. A known amount of OP was immersed in a ZnCl_2_ (3M) solution for 24 h and then heated to a temperature of 105 °C in an oven for 12 h [16]. The material thus prepared was labeled as AZOP. Different OP samples were immersed in a solution of H_3_PO_4_ (0.6 M) for 2 h and then dried at 95 °C for 12 h [30]. This material was labeled as AHOP. Thermochemical activation on the other hand was performed using ZnCl_2_ and H_3_PO_4_. For activation with ZnCl_2_, the OP sample was impregnated with ZnCl_2_ (3 M) under constant stirring (200 rpm) for 24 h. One part was taken to a muffle furnace and calcinated at 550 °C (called AZOP-550) and the other part at 600 °C (called AZOP-600). The calcination process for both samples lasted 30 min. Each sample was then immersed in an HCl solution (2 M) and stirred at 200 rpm for 3 h followed by a washing stage with distilled water up until a neutral pH was obtained. For thermochemical activation with H_3_PO_4_, the same chemical transformation procedure was carried out. Then, the sample was taken to the muffle furnace where the sample was heated at 600 °C for 3 h under nitrogen flowing at 150 cm^3^ min^−1^. This material was labeled as AHOP-600. Finally, all of the activated materials were vacuum filtered, dried in an oven at 105 °C for 12 h and macerated to a particle-size < 0.420 mm.

### 2.2. Physicochemical Characterization 

The physicochemical characterization of the materials was performed using different analysis techniques. In order to assess (OP) composition, the proximate analysis and elemental analysis were carried out. The surface area determination was performed by using the BET method (Brunauer, Emmett, and Teller) and the active functional groups were studied by Fourier transformed infrared spectroscopy (FT-IR) in the of 4000 to 450 cm^−1^ range using a Spectrum two-PerkinElmer with UATR. While the pH of zero point charge (pH_PZC_) was determined by the salt addition method [31] and, the acidic and basic surface functional groups of the materials were quantified using the method proposed by Boehm [32]. 

### 2.3. Adsoption Tests

#### 2.3.1. Batch Adsorption Studies 

In this study, the effects of pH and the adsorption time on adsorption performances were evaluated by using batch adsorption studies. Firstly, preliminary adsorption tests were performed to assess the performance of the different materials as follows: for each adsorbent material, a sample of 0.05 g was mixed in an Erlenmeyer flask with 50 mL of MB solutions with a concentration of 50 mg L^−1^ at natural pH (~7.4). The solution was stirred (200 rpm) for 2 h and the remaining concentration of MB in solution was determined using the spectrophotometer (VIS-DR 3900) absorption response at λ = 665 nm.

The MB removal percentage for each sample was calculated using Equation (1) and, the dye adsorption capacity of the materials was determined employing Equation (2).

(1) $$\%R=\frac{C_{0}-C_{t}}{C_{0}}\times 100$$

(2) $$q_{t}=\frac{C_{0}-C_{t}}{w}\times V$$

In these equations, *C*_0_ (mg L^−1^) corresponds to the initial concentration of MB in solution, *C_t_* (mg L^−1^) corresponds to MB concentration at time (t) and *q_t_* (mg g^−1^) means the amount of MB adsorbed per g of adsorbent material at time *t*, *V* (L) is the volume of the solution and *w* (g) represents the mass of the adsorbent material. From these tests, the materials with the best adsorption performance were selected for the kinetic and isothermal adsorption analysis. To evaluate the influence of pH on MB adsorption, a MB solution of 100 mg L^−1^ was adjusted at pH values of 2, 4, 6, 8, and 10 by using either NaOH or HCl (0.1 M) solutions and the adsorbent materials tests were OP, AZOP-550, and AHOP.

#### 2.3.2. Adsorption Isotherms and Kinetics

Batch experiments were conducted to evaluate the rate and adsorption equilibrium phenomena related to MB adsorption on OP, AZOP-550, and AHOP. A stock solution of 1000 mgL^−1^ MB was prepared and, the varying concentration of MB (50, 100, 150, 200, and 250 mg L^−1^) was prepared from the stock solution. After that, 500 mL of each solution were mixed with 0.5 g of the adsorbent material at natural pH and 25 °C. The mixture was stirred at 200 rpm with a rotating propeller and samples were taken at different times until the equilibrium conditions were reached. In order to eliminate dilution effects, it was verified in every experiment that the total subtracted volume did not exceeded 5% of the total volume [33]. 

All of the experimental data were adjusted to the pseudo-first, pseudo-second order, and the intra-particular diffusion kinetic models [34,35,36] following the equations shown in Table S1. The adsorption isotherm models used to fit the experimental data were Langmuir, Freunlich, Temkin, and Dubinin–Radush–Kevich models, which are presented in Table S2 [37,38,39,40].

### 2.4. DFT Calculations

The adsorption interactions between MB^+^ and the functional groups of the adsorbent materials were characterized through quantum chemical calculations. All of the calculations were performed with the Gaussian09 software package [41] using the DFT-B3LYP method with a basis set of 6–31+G(d,p). All of the geometry optimizations and the vibrational frequencies analyses were conducted by considering the solvent effect, which considers long-range implicit hydration by applying the integral equation formalism variant of the polarized continuum model (IEFPCM) [42]. The local minimum potential energy values for each structure were confirmed since imaginary frequencies did not occur. Simulations of adsorbent materials surfaces with different oxygenated functional groups were conducted based on the experimental results. The adsorption energy (E_ads_) of MB^+^ on the carbonaceous model was determined by using Equation (3):

(3) $$E_{\mathrm{ads}\;}{=E}_{\left(\mathrm{MB}+\right)-\mathrm{surface}}{\;-\;(E}_{\left(\mathrm{MB}+\right)}{+E}_{\mathrm{surface}})$$

where $E_{\left(\mathrm{MB}+\right)-\mathrm{surface}}$ is the total energy of the complex formed by MB^+^ and the carbonaceous surface. $E_{\left(\mathrm{MB}+\right)}$ corresponds to the total energy of the MB^+^ molecule, and $E_{\mathrm{surface}}$ is the total energy of the carbonaceous model.

## 3. Results and Discussion

### 3.1. Performance of Adsorbent Material on MB Removal: Preliminary Tests

**Figure 2:**
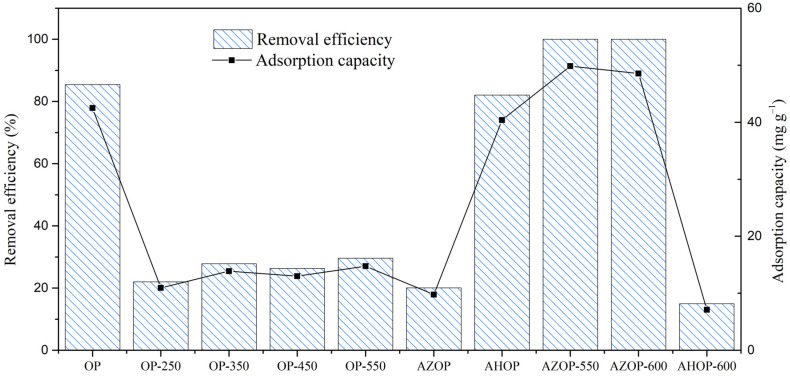
Comparison of MB removal efficiency and adsorption capacity for the different adsorbent materials.

To assess which materials, have the best adsorption performance, preliminary MB adsorption experiments were carried out on all of the adsorbents (Figure 2). 

For materials produced from thermal transformation of biomass (OP-250, OP-350, OP-450, and OP-550), low removal capacities can be observed; moreover, there is no significant effect of biomass calcination temperature on the MB removal capacity, with values between 10.95 and 14.75 mg g^−1^ for OP-250 and OP-550, respectively. 

For AZOP and AHOP materials produced by chemical transformation, it is evidenced that AHOP is five-times better in terms of removal efficiency and adsorption capacity respect to AZOP. However, when AHOP is compared with OP, very similar behavior can be evidenced. It should be that the addition of phosphoric acid, does not modify the oxygenated groups on the material surface, and therefore there is no change in the active groups for the adsorption of MB [17]. Whereas, when ZnCl_2_ is used, it is evident that there is a blockage of the active groups for the adsorption of MB, which are present in OP.

The materials produced from the thermochemical modification showed that the materials modified with ZnCl_2_ are more efficient for the removal of MB in comparison to the ones activated with H_3_PO_4_. For AHOP-600 only a removal of 16% of MB was observed. It is probably due to carbonization at high temperatures where surface functional groups that contain oxygen are released, limiting the number of active sites for MB adsorption [36]. While it has been reported that for carbonaceous materials activated with ZnCl_2_, high temperatures favor the formation of porosity generated by the dehydrating nature of this activating agent. With this observation, it is possible to deduce that MB adsorption occurs both by interaction with oxygenated functional groups and by porosity. 

However, at this point, it is not possible to identify which of these two ways of adsorption is the one that contributes the most to the removal of MB by OP, and AZOP-550. For this, it was necessary to carry out a detailed textural analysis and chemical properties of the surface functional groups of these materials. Table 1 and Table 2, show that for OP, AHOP, and AZOP-550, the materials with the highest MB removal efficiency there is a great difference in textural and chemical properties. For instance, OP and AHOP-600 are materials with low surface area (3 and 7 m^2^ g^−1^, respectively), while AZOP-550 is a material with very good porosity development (1078 m^2^ g^−1^). In this way, while the adsorption capacity of AZOP-550 is about 15% larger than that of the other two materials (OP and AHOP) (see Figure 2), the corresponding surface area is about 350- and 180-times larger than those of OP and AHOP, respectively. It suggested that the interaction with functional groups is the main path of MB adsorption on these materials.

**Figure 3:**
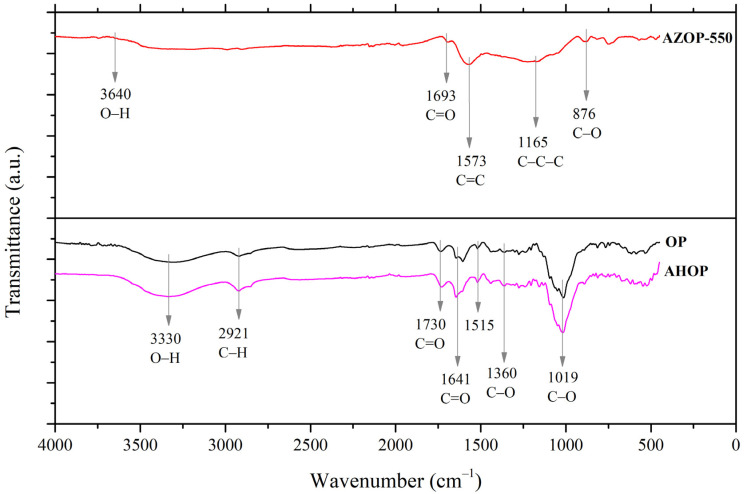
FTIR Spectra of OP, AHOP, and AZOP-550 adsorbents.

The effect of OP treatment on surface morphology (area and pore volume) should therefore be coupled to chemical changes on the surface functional groups that define MB-adsorbent surface intermolecular interactions and therefore, the adsorption capacity of the materials. Figure 3 shows the FTIR spectra for OP, AHOP, and AZOP-550 adsorbents. While OP and AHOP show very similar spectra, the IR response for AZOP-550 is characterized by noticeable changes that are related to the degradation of functional groups and breaking of chemical bonds due to the high temperature of thermochemical process [43]. The bands in OP and AHOP show a signal at 3330 cm^−1^ that is related to the O–H stretch mode of the hemicellulose and the signal at 2921 cm^−1^ that is associated with the C–H elongation vibrations of lignin. The presence of C=O at 1730 cm^−1^ is attributed to the ketone or aldehyde groups of hemicellulose and the signal located at 1641 cm^−1^ is related to carboxylic acid moieties. The band at 1515 cm^−1^, is associated to the vibration of the lignin aromatic ring, the signal at 1360 cm^−1^ is attributed to the stretching of the C–O bonds of carboxylic acids present in cellulose, hemicellulose, and lignin groups and the peak around 1019 cm^−1^ may be related to the elongation of the vibration of C–O bonds in phenols, ethers, and alcohols [23,43]. In the case of the spectrum for the thermochemically modified material, AZOP-550, the signals at 3640, 1693, 1573, 1165, and 876 cm^−1^ are related to the presence of O–H, C=O, C=C, bending of the C–C–C, and the C–O [23,44,45].

From Table 2, it can be observed that OP and AHOP are the materials with the highest amount of oxygenated functional groups, being 1.8- and 2.3-times higher with respect to AZOP-500, respectively.

The reduction of the total number of acidic groups (lactone and phenol groups) on the surface of AZOP-550, can be explained by the decomposition reactions of the cellulose, hemicellulose, and pectin—the main components of the OP biomass [43]. The larger concentration of acidic surface functional groups on OP and AHOP should be playing an important role in the selectivity and adsorption capacity for MB removal [46]. In these cases, there is less surface adsorption area than that of AZOP-550, but there is a larger density of acidic groups that, depending on the protonation state, should explain the behavior of adsorption interaction sites. Therefore, depending on the pH, different types of interactions take place between MB and the adsorbent materials.

### 3.2. pH Effect on MB Adsorption 

**Figure 4:**
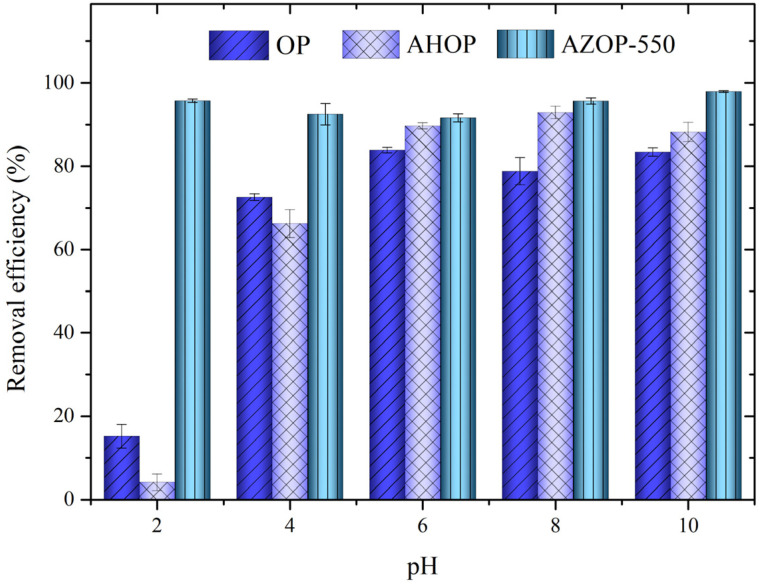
Effect of pH in the MB adsorption for the different substrates under study.

The pH value of the solution is an important parameter to control the adsorption process, since it may affect MB adsorption by changing the surface charge of the adsorbents and ionization behavior of adsorbents and dye [47]. Figure 4 shows the influence of pH on MB adsorption for OP, AHOP, and AZOP-550. At pH 2, the MB removal efficiency is lower for OP and AHOP, than for AZOP-550. At pH values higher than 4, MB removal increases for OP and AHOP and remains roughly constant for the AZOP-550 material. These results suggest that there is a common pH dependence for OP and AHOP that is not observed for AZOP-550. To understand the pH influence, it is necessary considering the pH_(PZC)_ of the adsorbents and the pK_a_ (3.8) for MB [48]. For MB, pH values above 3.8, the cationic species are the preponderant MB species in the solutions [49]. For adsorbents, Table 2 shows that OP and AHOP have pH_(PZC)_ values of 3.5 and 3.4, respectively, while AZOP has a value of 6.3 (see Figure S2 from Supplementary Materials). Similar observations have been reported in the literature, where M. Boumediene et al. [50] obtained a pH_(PZC)_ of 3.8 for orange peel without any transformation and A. Guediri et al. [51] transformed the orange peel using H_3_PO_4_ and obtained a pH_(PZC)_ of 3.34. This observation suggests that at a pH value of 2, OP, AHOP, and MB are positively charged and therefore the low dye removal percentages. 

At pH values above the pH_(PZC)_ the adsorbent materials are negatively charged, favoring the cationic MB adsorption through electrostatic interactions. On the other hand, the pH does not have a significant effect for MB adsorption over AZOP-550, indicating that for the AZOP-550 material the high surface area and porosity are the main factors that control the MB adsorption process.

### 3.3. Adsorption Kinetics

Table S4 shows the kinetic parameters obtained from the fitting of the experimental data to the pseudo-first, pseudo-second order, and to the intra-particular diffusion models. The results show values of R^2^ = 0.999 for the pseudo-second order indicating that this model is a good representation of the MB adsorption process. Since this model considers the dependence of the adsorption rate on two concentrations, it can be assumed that chemisorption is the controlling step of the MB adsorption process over OP, AHOP, and AZOP-550.

To identify the reaction adsorption pathways and mechanisms that operate in these processes, the intra-particle diffusion model was employed. Table S4 shows a good fit to this model for MB removal on OP, AHOP, and AZOP-550, where the values of k_1d_ are larger than the ones of k_2d_, suggesting that surface adsorption is faster than the adsorption inside the pores. C_2_ values are larger than the values of C_1_, indicate that the contribution of adsorption on the surface when compared to that taking place in the pores is more important. These results are consistent with reports of MB removal using different adsorbents produce from biomass [24,50,51]. It is evident that MB adsorption on all of the adsorbent materials is a complex process involving multiple mechanisms (adsorption on the surface and diffusion within the pores of the adsorbent) for which the analysis of equilibrium adsorption data could shed some light. 

### 3.4. Adsorption Isotherms

Four isotherm models were employed to assess the adsorption of MB on the adsorbent materials at equilibrium. According to the R^2^ values (Table 3), all of the models fitted the experimental data very well indicating a complex adsorption equilibrium process for all materials that partially follows the mechanisms that define the models under consideration. From Table 3, $R_{L}$ value for all adsorbent materials falls in the range 0 < $R_{L}$ < 1 suggesting that the adsorption process is in all cases favorable. Fitting of the experimental data to the Langmuir adsorption model also reveals that the maximum adsorption capacities $(Q_{m})\;$ for OP, AHOP, and AZOP-550, correspond to 192.31, 277.78, and 232.56 mg g^−1^, respectively. The fact that $(Q_{m})\;$ is in the same order of magnitude for the three adsorbent materials, that is, a similar active adsorption surface area, is consistent with a picture of relatively low area and optimized MB adsorption chemical surface structure for OP and AHOP, and a large surface and poorly chemically structured surface for AZOP-550 (see Figure S4 from Supplementary Materials).

As can be seen in Table 3, the fitting of the experimental adsorption data to the Freundlich model also suggests that MB adsorption takes place by a mixture of interactions with the different functional groups of the adsorbent surface; involving as expected, a variety of specific interaction energies. In addition, the n values that were found from fitting of the experimental data to the Freundlich model, are larger than 1, indicating that adsorption corresponds to a physical process. This observation is supported by the fitted b values < 8 kJ mol^−1^ obtained from the Temkin model, which are characterized by a physical adsorption processes, that results from van der Waals type weak interactions [52]. The weak physical nature of the interaction of MB with the OP, AHOP, or AZOP-550 adsorbent materials, is further supported by the E value (average adsorption energy) obtained from the Dubinin–Radushkevich model (E < 8 kJ mol^−1^) [53].

### 3.5. Adsorption Mechanism

**Figure 5:**
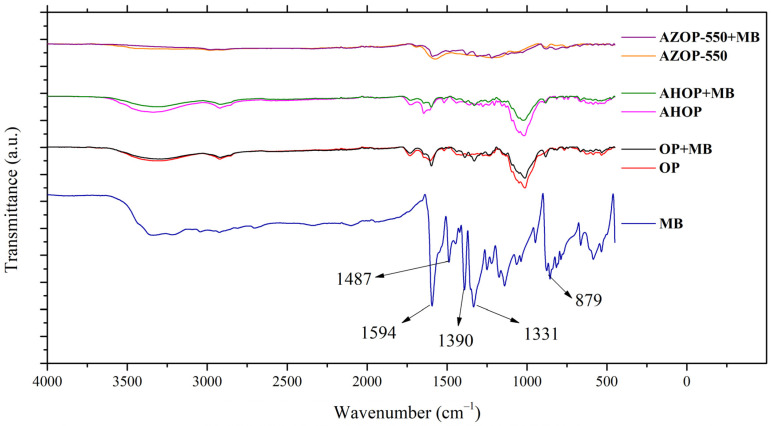
FTIR Spectra of the biochar materials under study before and after MB adsorption.

Figure 5 shows the spectra of MB before and after adsorption on OP, AHOP, and AZOP-550. The characteristic bands of methylene blue are: 1594 cm^−1^, which corresponds to the vibration of C–N at 1487 cm^−1^, related to the C–C bond of the aromatic ring, 1390 cm^−1^, associated to the vibration of C–N in the –N(CH_3_)_2_^+^ group, 1331 cm^−1^, related to the –CH_3_ group and 879 cm^−1^ corresponding to the C–H bond in the aromatic ring [54]. After MB adsorption, shifts in the IR bands are observed. For instance, in OP and AHOP adsorbents, the band at 1605 cm^−1^ shifts to 1594 cm^−1^ (C–N), the band at 1484 cm^−1^ is slightly displaced to 1487 cm^−1^ (C–C), and the signal at 890 cm^−1^ shifts to 879 cm^−1^ (C–H). While these shifts may reflect π–π interactions in the adsorption process, electrostatic interactions should be reflected by the shift of the band (from 1402 to 1390 cm^−1^) which is associated to the functional group containing the positively charged N as well as by the symmetrical deformation of –CH_3_ which shifts from 1323 to 1331 cm^−1^ [55]. For AZOP-550, IR band shifts of the C–N and C–C bonds of the aromatic rings (from 1574 and 1470 cm^−1^ to 1594 and 1487 cm^−1^, respectively) suggest that the main dye adsorption force for this substrate, corresponds to π–π interactions.

**Figure 6:**
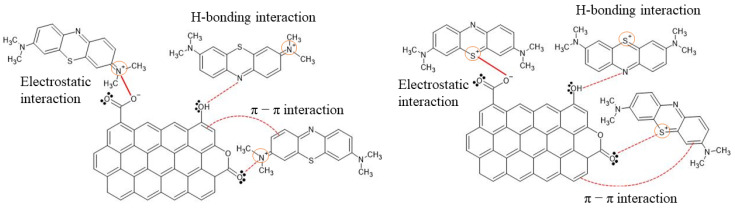
Scheme of the adsorption interactions of MB on biochar materials.

From the analysis of the experimental data previously discussed, it is possible to suggest that MB adsorption on OP, AHOP, and AZOP-550 is taking place by a mixture of the following interactions: (i) hydrogen bonds (interaction between the N (nitrogen) of the MB structure and an OH group on the surface of the adsorbent material), (ii) π–π interactions between the aromatic structure of the material and the benzenic rings of MB, and iii) electrostatic interactions, which are presented at pH > 4, that is, above the pH_pzc_ of the materials and the pKa of the dye, the MB dye is a cationic species capable of interacting with the negatively charged surface of OP and AHOP (for AZOP-550, surface deprotonation takes at pH values higher than 6.3). These observations are consistent with previous studies on MB adsorption on the surface of different adsorbents produced from biomass [4,10,45], Figure 6 shows a schematic representation of the interactions that should be taking place during the MB adsorption event. 

**Figure 7:**
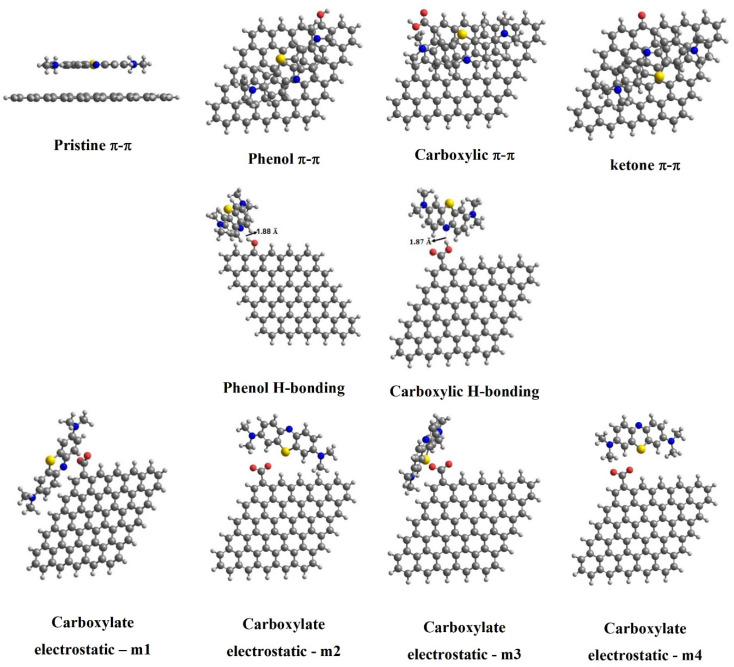
The optimized structures of MB^+^ adsorption on oxygenated and pristine carbon surfaces.

The characterization of the interactions between MB^+^ and the adsorbent materials, was carried out through theoretical calculations. As it was previously discussed, in this work, three adsorption mechanisms have been considered: (i) π–π stacking interactions, (ii) electrostatic interactions, and (iii) surface complexation reactions (H–bonding) with the chemical moiety (amino) of MB^+^ and the (–OH) group on the adsorbent surface. Figure 7 shows visual illustrations of these interactions and the corresponding adsorption energies are provided in Table 4.

From these results, it can be concluded that: (i) in all cases, an adsorbent surface containing oxygenated functional groups is characterized by higher adsorption energies with MB than a surface without these functional groups, (the ketone group constitutes an exception); (ii) the aromatic rings of MB+ favor π–π stacking interactions, (iii) strong MB^+^-carbon surface interactions are due to electrostatic effects while weak MB^+^–carbon surface interactions are due to π–π stacking phenomena, (iv) the sequence of maximum adsorption energy is electrostatic (−8.46 kcal/mol) > H–bonding (−6.44 kcal/mol) > π–π stacking interactions (−0.50 kcal/mol). This agrees with the experimental results, where it was found that at pH values above the pH_pzc_, the surface is negatively charged and therefore, the highest adsorption of MB^+^ takes place.

On the other hand, AZOP-550 may be characterized by adsorption from diffusion in the pores of the adsorbent due to the high surface area of this material (1078.56 m^2^ g^−1^). This interpretation is supported by the higher R^2^ value obtained from fitting the experimental data to the intraparticular diffusion model for AZOP-550 when compared to the values computed for OP and AHOP. 

### 3.6. Comparison with Other Adsorbents

Table 5 shows the comparison of the maximum adsorption capacity (Q_m_) of MB on OP, AHOP, and AZOP-550 with some recent reports dealing with OP-based adsorbent materials. Inspection of the content of Table 5 shows that residues from orange peel have a great potential to produce efficient adsorbent materials for MB removal in aqueous solutions. The adsorbents used in this work were produced using simple methodologies, which implies low costs in their production. It was also reported that the orange peel without any transformation has potential for application in water. Therefore, the adsorbents reported in this work can become a cost-effective alternative for reducing MB by adsorption.

## 4. Conclusions 

The results found in this study show that by using different transformations, it is not only possible to obtain adsorbent materials from orange peel residues, but that these materials are effective for MB removal. The existence of oxygenated surface functional groups on the obtained adsorbents (such as –OH, –COO^−^, –COOH, and –CO) allows for specific pH values that guarantee the deprotonation of surface oxygenated groups by interactions with the cationic dye MB in aqueous solution. This effect is compensated with large adsorbent areas that can be obtained by thermochemical treatment to generate partially oxygenated adsorbent biochar. The kinetic and isothermal parameters on the other hand, allowed one to conclude that the adsorption process involves a mixture of both physical and chemical interactions, obtaining high adsorption capabilities for MB. Based on DFT calculations and FTIR analysis before and after MB adsorption, it can be concluded that the main mechanisms that exist in the dye adsorption process on the different adsorbent materials are: (i) electrostatic interaction between the positive charge of the MB molecule and the negative charge of the adsorbent surface due to oxygenated functional groups; (ii) surface complexation reactions (H–bonding) among the functionality (amino) of the MB^+^ with (–OH) of the oxygenated groups on carbon surface, and (iii) π (MB)–π (biochar) interactions.

## Figures and Tables

**Table 1:** Textural properties of the adsorbent materials under study.

Material	Surface Area BET (m^2^ g^−1^)	Pore Volume (cm^3^ g^−1^)
OP	3.098	0.0013
AHOP	7.0142	0.0030
AZOP-550	1078.56	0.5205

**Table 2:** Content of surface chemical groups for the adsorbent materials under study.

Material	Carboxylic Group (mmol g^−1^)	Lactone Group (mmol g^−1^)	Phenol Group (mmol g^−1^)	Total Acid Sites (mmol g^−1^)	Total Basic Sites (mmol g^−1^)	pH _(PZC)_
OP	0.650	0.925	0.200	1.774	0.000	3.5 ± 0.08
AHOP	0.925	1.325	-	2.250	0.125	3.4 ± 0.11
AZOP-550	0.775	0.150	0.050	0.975	0.225	6.3 ± 0.03

**Table 3:** Parameters for the different isotherm adsorption models for the adsorption of MB on OP, AZOP-550, and AHOP.

Material	OP	AHOP	AZOP-550
**Langmuir**			
**Q_m_ (mg g^−1^)**	192.31	277.78	232.56
**K_L_ (L mg^−1^)**	0.03	0.02	0.54
**R_L_**	0.40–0.11	0.48–0.16	0.4–0.01
**R^2^**	0.97	0.98	1.00
**Freundlich**			
**K_F_ (mg g^−1^)**	14.17	16.24	83.60
**n**	1.86	1.87	3.03
**R^2^**	0.85	0.98	0.73
**Temkin**			
**K_T_ (L g^−1^)**	0.31	4.39	9.12
**b (kJ mol^−1^)**	0.06	0.04	0.06
**R^2^**	0.96	0.98	0.89
**Dubinin–Radushkevich**			
**K_DR_ (mol^2^ kJ^−2^)**	20.39	18.83	0.31
**q_s_ (mg g^−1^)**	134	166	206
**E (KJ mol^−1^)**	0.16	0.16	1.28
**R^2^**	0.98	0.86	0.94

**Table 4:** Adsorption energy values (kcal mol^−1^) for the interactions between methylene blue with a pristine carbon surface and different oxygen functional groups. (The total energy (Hartree) of all systems are presented in the Table S5.).

Interactions	Pristine	**Functional Groups**			
		Phenol (–OH)	Carboxylate (–COO^−^)	Carboxylic (–COOH)	Ketone (–CO)
	Adsorption Energy (kcal mol^−1^)				
(π–π)	0.20	−0.50		−0.25	1.61
(H–bonding)		−5.76		−6.44	
Electrostatic			−3.60 (m1) −8.46 (m2) −8.08 (m3) −7.95 (m4)		

**Table 5:** Comparison of maximum adsorption capacities of MB on different adsorbents.

Material	Treatment	pH	Q_m_ (mg g^−1^)	Reference
OP	Washed biomass	pH natural (4.5)	192.31	This study
AZOP-550	Thermochemical activation using ZnCl_2_ (550 °C)	pH natural (7.5)	232.56	This study
AHOP	Chemical activation using H_3_PO_4_	pH natural (3.7)	277.78	This study
ZnCl_2_-AC	Thermochemical activation using ZnCl_2_ (800 °C)	7–8	281.52	[24]
OP	Washed biomass	4	14.16	[21]
SOP	Chemical activation using NaOH	9	18.28	[21]
OP	Washed biomass	pH natural (4.2)	218	[19]
OP-H_3_PO_4_	Chemical activation using H_3_PO_4_	pH natural (6.2)	307.63	[51]
COP 400 °C	Calcination a 400 °C	4.98	14.85	[18]
OP-ZnCl_2_	Chemical activation using ZnCl_2_	9	7.57	[20]

## Data Availability

The datasets used and/or analyzed during the current study are available from the corresponding author on reasonable request.

## Supplementary Materials

The following are available online. Figure S1 FTIR spectra of calcined materials at different temperatures. Figure S2 pH PZC of adsorbent materials. A: OP; B: AZOP-550; C: AHOP. Figure S3 Graphs of the adsorbents (OP, AHOP and AZOP-550) for kinetic models with the equation (C0 50 mg L^−1^). Figure S4 Graphs of the adsorbents (OP, AHOP and AZOP-550) for isotherm models with the equation. Table S1 Equation kinetic models. Table S2 Equations models isotherms. Table S3 Biomass analysis data. Table S4 Parameters for the different kinetic models for the adsorption of MB on OP, AZOP-550 and AHOP. Table S5. Total energy (Hartree) of all systems
