# Supplementary material for: Experimental and Theoretical Insights on Methylene Blue Removal from Wastewater Using an Adsorbent Obtained from the Residues of the Orange Industry
Source: Molecules. 2021 Jul 28;26(15):4555. doi: 10.3390/molecules26154555 (PMC8348386; doi:10.3390/molecules26154555)
Supplement: Supplementary file 1 [file molecules-26-04555-s001.zip › molecules-1257784-supplementary.pdf]

Supplementary data

Experimental and theoretical insights on methylene blue removal from wastewater using an adsorbent obtained from the residues of the orange industry

Stephanie Giraldo[a], Irma Robles[b], Luis A. Godínez[b], Nancy Acelas[a*], Elizabeth Flórez[a*]

[a] Grupo de investigación Materiales con Impacto (Mat&mpac), Facultad de Ciencias Básicas, Universidad de Medellín, Carrera 87 No. 30-65, Medellín, Colombia

[b] Centro de Investigación y Desarrollo Tecnológico en Electroquímica S. C., Parque Tecnológico Querétaro, 76703 Sanfandila, Pedro Escobedo, Querétaro, Mexico

[*]Corresponding author

E-mail address: nyacelas@udem.edu.co (N Acelas), elflorez@udem.edu.co (E Flórez)

Table S1 Equation kinetic models.

Pseudo-first order	$Log(q_e - q_t) = Log\ q_e - \frac{k_1}{2.303}t$ (1)
Pseudo-second order	$\frac{t}{q_t} = \frac{1}{k_2\ q_e^2} + \frac{t}{q_e}$ (2)
Intraparticle diffusion	$q_t = k_{di}\sqrt{t} + C_i$ (3)

q_t: amount of MB adsorbed in a time t (mg g^{-1}); q_e amount of MB adsorbed at equilibrium (mg g^{-1}); t: time (min); k_1: pseudo first order adsorption rate constant (min^{-1}); k_2: constant speed of pseudo second order (g mg^{-1} min^{-1}); k_{di} : Intraparticle diffusion rate constant (mg g^{-1} $min^{-1/2}$); C_i: constant (mg g^{-1}).

Table S2 Equations models isotherms.

Langmuir	$\frac{C_e}{q_e} = \frac{1}{Q_m K_L} + \frac{C_e}{Q_m}$ (1)
Freundlich	$LogQ_e = LogK_F + \frac{1}{n} LogC_e$ (2)
Non-dimensional separation factor	$R_L = \frac{1}{1 + K_L C_i}$ (3)
Temkin	$q_e = \frac{RT}{b} LnK_T + \frac{RT}{b} LnC_e$ (4)
Dubinin–Radushkevich	$Ln\ (q_e) = Ln(q_s) - K_{ad}\ \varepsilon^2$ (5)
Constante de Dubinin–Radushkevich	$\varepsilon = RTln\left(1 + \frac{1}{C_e}\right)$ (6)
Free energy	$E = \frac{1}{\sqrt{2K_{ad}}}$ (7)

C_e : MB concentration in equilibrium (mg L^{-1}); q_e: amount of MB adsorbed at equilibrium (mg g^{-1}); Q_m: maximum capacity of adsorbate (mg g^{-1}); K_L: constant of Langmuir (L g^{-1}); K_f: Freundlich dissociation constant (mg g^{-1}); n: constant related to reaction intensity; R_L: non-dimensional separation factor; C_i: is the initial concentration of MB (mg L^{-1}); b: constant associated with the heat of adsorption (kJ mol^{-1}); K_T: Temkin constant (L g^{-1}); T: temperature (K); R: gas constant (0.008314 kJ mol^{-1} K^{-1}); q_s: theoretical capacity of isothermal saturation (mg g^{-1}), k_{ad}: isothermal constant of Dubinin–Radushkevich (mol^2 kJ^{-2}); ε: Dubinin–Radushkevich constant; E: Free energy (KJ mol^{-1}).

Table S3 shows the data corresponding to the proximate analysis and elemental analysis of biomass (OP). High volatile material content (87.15%) and average fixed carbon content (8.90%) were found, indicating that a reasonable amount of carbon is available for thermochemical transformation. In addition, the high elemental content of carbon (43.60%) indicates that OP is a suitable precursor for the preparation of adsorbent materials.

Table S3 Biomass analysis data

Biomass	Moisture Content (%)	Proximate Analysis (Wt %)[a]			Elemental Analysis (Wt %)[a]				
		MV	CF	Ashes	N	C	H	S	O[b]
OP	7.42	87.15	8.90	3.95	0.60	43.60	5.60	0.20	50.00

MV: Volatile material; CF: Fixed Carbon; N: Nitrogen; C: carbon; H: Hydrogen; S: Sulfur; O: Oxygen

OP: Orange Peel Biomass

[a] On a dry basis   [b] By difference (O% = 100% - C% - N% - H% - S%)

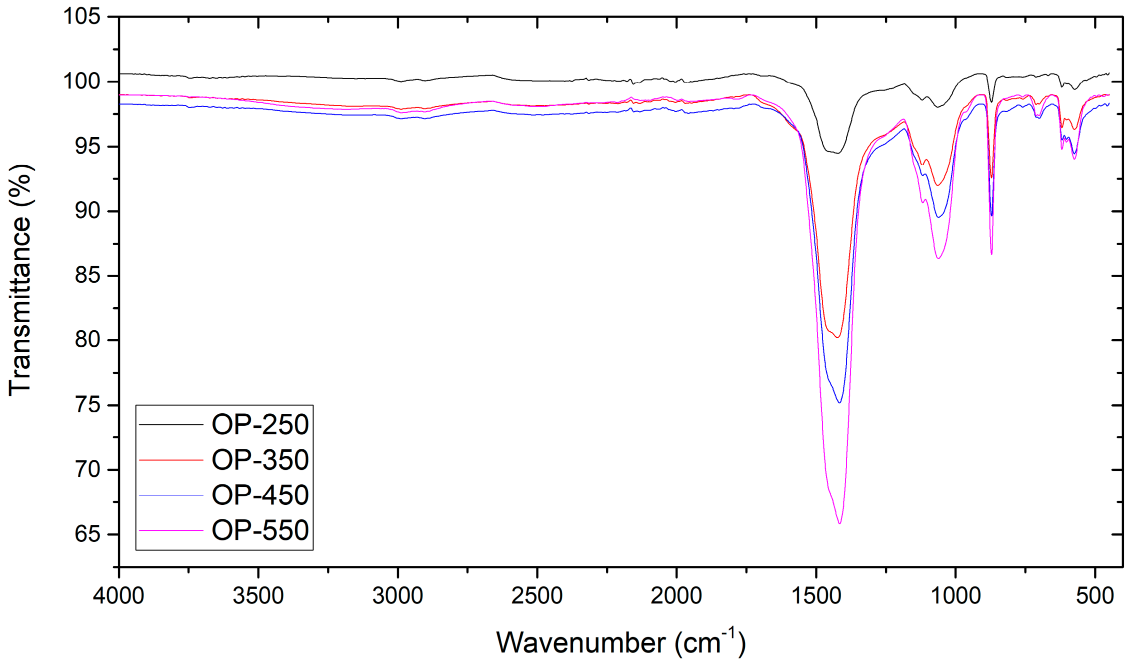

Figure S1 FTIR spectra of calcined materials at different temperatures.

Table S4 Parameters for the different kinetic models for the adsorption of MB on OP, AZOP-550 and AHOP.

**Material**	**OP**					**AZOP-550**					**AHOP**				
**C_0 (mg L^{-1})**	**50**	**100**	**150**	**200**	**250**	**50**	**100**	**150**	**200**	**250**	**50**	**100**	**150**	**200**	**250**
**$q_{e\ exp}$ (mg g^{-1})**	35.35	83.35	107.51	138.40	146.26	48.89	100.08	141.52	180.59	190.19	41.80	77.05	87.84	96.37	108.14
**Pseudo first order**															
**k_1 (min^{-1}) x 10^{-2}**	0.18	0.16	0.81	0.60	0.16	5.99	0.94	0.35	0.28	0.02	4.63	2.67	3.20	4.35	4.42
**q_e (mg g^{-1})**	11.02	16.63	26.28	34.15	31.56	22.79	36.39	63.56	109.67	119.67	19.69	38.67	42.78	49.42	55.74
**R^2**	0.68	0.45	0.67	0.61	0.43	0.96	0.82	0.87	0.95	0.92	0.92	0.92	0.93	0.95	0.88
**Pseudo second order**															
**k_2 (g mg^{-1} min^{-1}) x 10^{-2}**	0.18	0.09	0.07	0.05	0.06	1.33	0.13	0.03	0.01	0.01	-	-	-	-	-
**q_e (mg g^{-1})**	34.72	84.03	111.11	138.89	147.06	49.26	101.01	142.86	185.19	222.22	40.98	72.46	84.75	94.34	109.89
**R^2**	1.00	1.00	1.00	1.00	1.00	1.00	1.00	1.00	1.00	1.00	1.00	1.00	1.00	1.00	1.00
**Intra-particle Diffusion**															
**K_{d1} (mg g^{-1} $min^{1/2}$)**	6.04	15.68	16.83	19.27	21.08	10.30	15.50	13.07	13.11	13.80	8.77	13.90	17.11	19.43	21.11
**C_1 (mg g^{-1})**	3.31	11.77	14.58	27.88	26.36	5.36	4.67	11.80	10.83	11.85	4.49	6.49	7.01	8.80	10.21
**R^2**	0.89	0.81	0.86	0.78	0.82	0.90	0.96	0.90	0.91	0.91	0.90	0.92	0.93	0.92	0.91
**K_{d2} (mg g^{-1} $min^{1/2}$)**	0.60	0.67	1.09	0.68	0.89	0.54	3.89	4.12	4.82	5.37	0.69	1.51	1.51	1.13	1.96
**C_2 (mg g^{-1})**	22.47	66.23	88.19	119.95	120.20	43.90	54.25	63.29	61.27	58.71	34.85	58.13	69.40	82.85	87.21
**R^2**	0.98	0.91	0.97	0.73	0.78	0.85	0.95	0.95	0.98	0.98	0.83	0.88	0.81	0.77	0.81

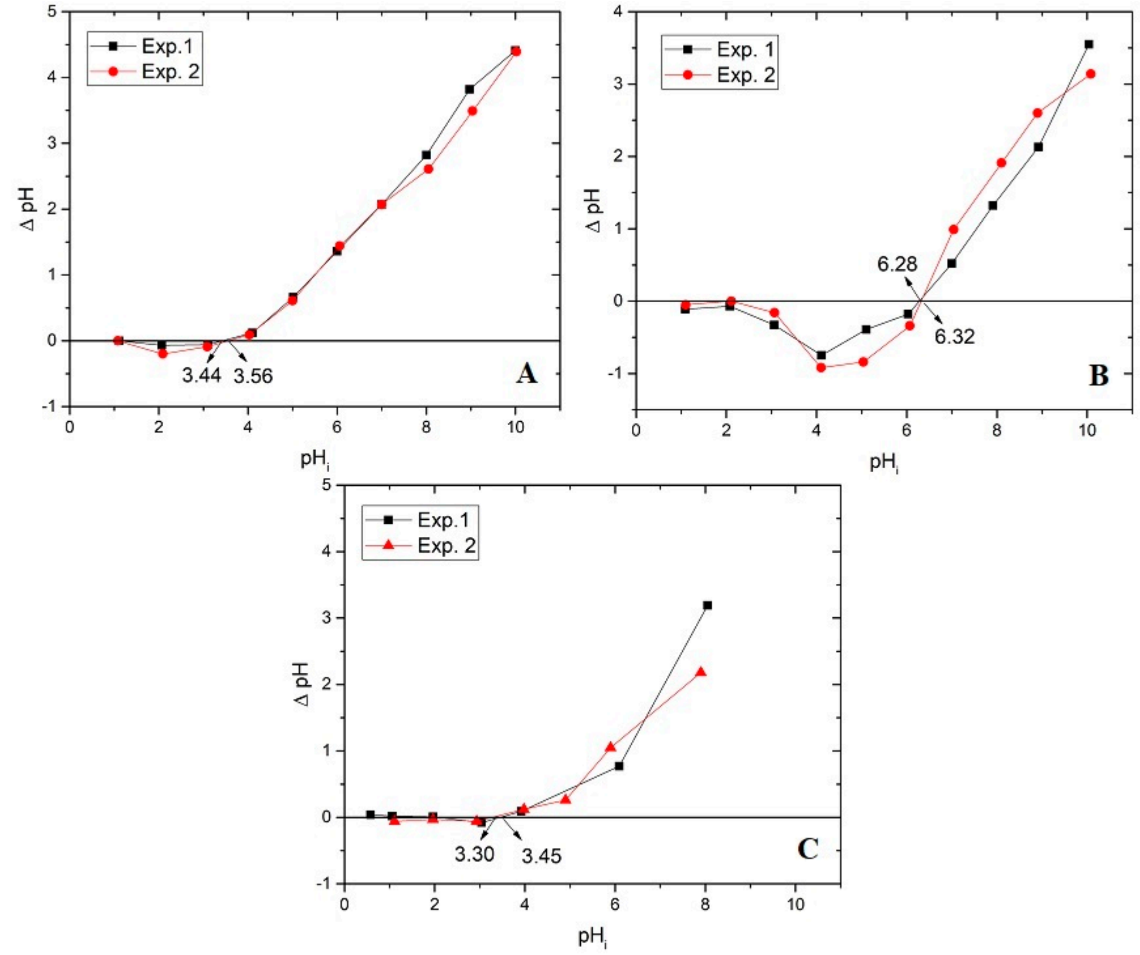

Figure S2 pH $_{PZC}$ of adsorbent materials. A: OP; B: AZOP-550; C: AHOP.

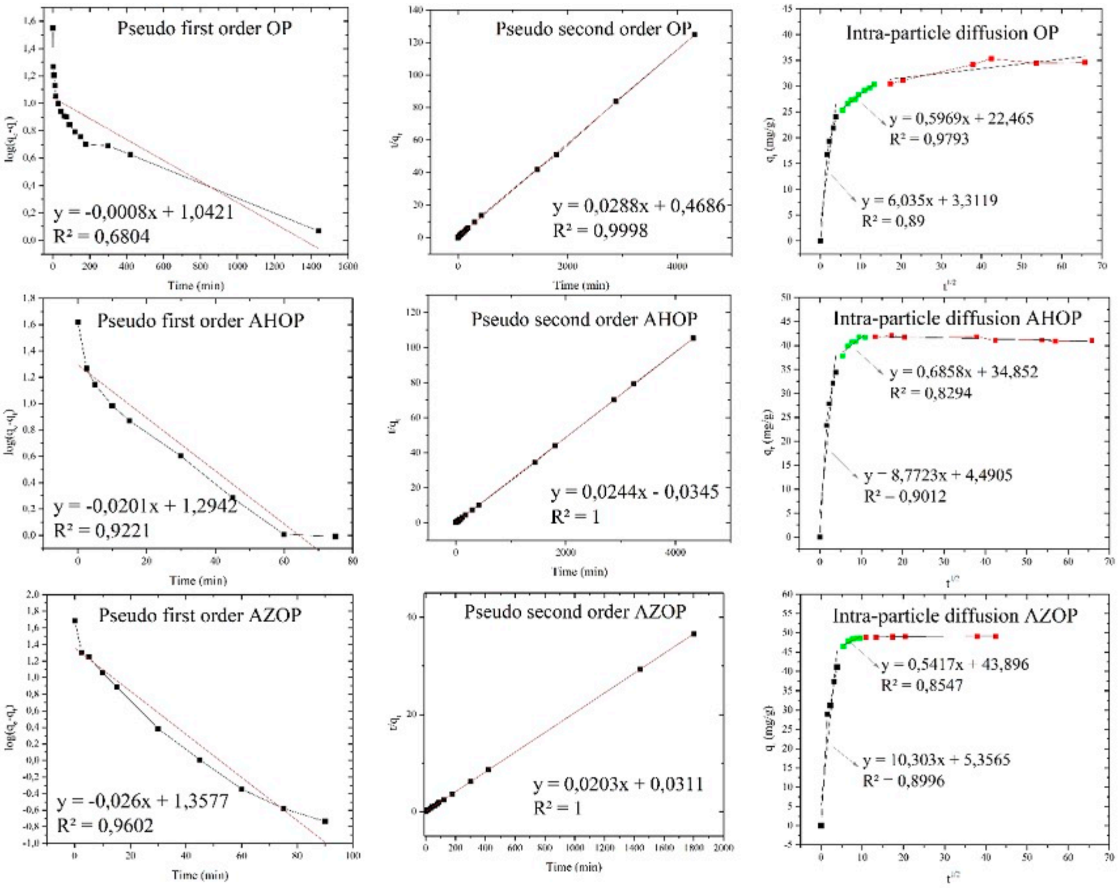

Figure S3 Graphs of the adsorbents (OP, AHOP and AZOP-550) for kinetic models with the equation (C_0 50 mg L^{-1}).

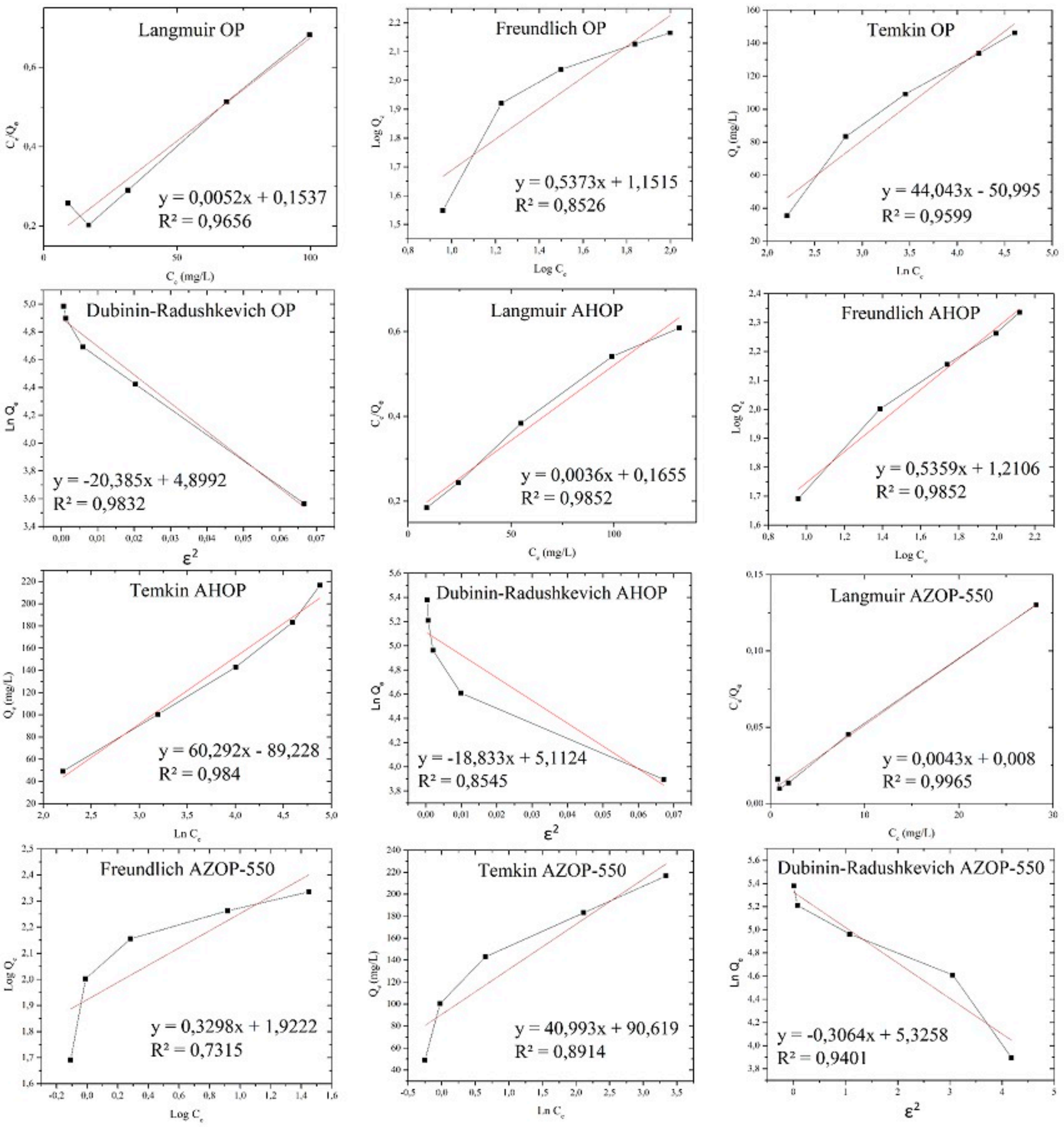

Fig. S4 Graphs of the adsorbents (OP, AHOP and AZOP-550) for isotherm models with the equation.

Table S5. Total energy (Hartree) of all systems

System	Total energy
**Pristine**	-2680.464443
**MB+**	-1182.544357
Pristine_MB+ (π-π)	-3863.008475
**Phenol (-OH)**	-2755.682871
Ar-OH _MB+ (π-π)	-3938.22802
Ar-OH _MB+ (H-bonding)	-3938.236415
**Ar-COOH**	-2869.023318
Ar-COOH_MB+ (π-π)	-4051.568077
Ar-COOH_MB+ (H-bonding)	-4051.577932
**Ar-COO-**	-2868.555377
Ar-COO-MB+ (electrostatic-m1)	-4051.105465
Ar-COO-MB+ (electrostatic-m2)	-4051.113211
Ar-COO-MB+ (electrostatic-m3)	-4051.112613
Ar-COO-MB+ (electrostatic-m4)	-4051.112396
**Ar-CO**	-2755.104225
Ar-CO-MB+ (π-π)	-3937.646012
